# Energetic Value of Women's Work: Assessing Maternal Energetic Costs From Acorn Foraging

**DOI:** 10.1002/ajpa.70023

**Published:** 2025-03-19

**Authors:** Alexandra Niclou, Alexandra Greenwald, Cara Ocobock

**Affiliations:** ^1^ Pennington Biomedical Research Center Baton Rouge Louisiana USA; ^2^ Department of Anthropology University of Notre Dame Notre Dame Indiana USA; ^3^ Department of Anthropology University of Utah Salt Lake City Utah USA; ^4^ Natural History Museum of Utah Salt Lake City Utah USA; ^5^ Eck Institute for Global Health University of Notre Dame Notre Dame Indiana USA

**Keywords:** energy expenditure, foraging, physical activity levels

## Abstract

**Objectives:**

Perceptions of female energetic contributions and their role in human evolution are limited. This exploratory study compares energy expenditure, return rate, and foraging efficiency between infant carrying methods in females simulating acorn foraging practices by Indigenous communities in western North America.

**Materials and Methods:**

After resting metabolic rate (RMR) was collected, female volunteers (*n* = 6, age: 21–37) conducted three 1‐h bouts of acorn foraging. First, volunteers foraged unloaded (control) while for the second and third bouts they foraged carrying a traditional basketry cradle or a chest sling (randomized order) with 4.5 kg sandbags. Energy expenditure (EE) was measured through indirect calorimetry, physical activity intensity was assessed using accelerometry, and foraging return rates (RR) were calculated after acorn processing.

**Results:**

The inter‐bout results are not statistically significant. Findings show, however, that foraging RR largely surpasses EE irrespective of infant carrying method. The cradle carrying technique resulted in the largest mean EE, yet it was more efficient than the sling‐carrying method. Most of the time foraging was spent at moderate physical intensity, especially during cradle‐carrying bouts compared to the sling‐carrying and control groups.

**Conclusions:**

This small exploratory study demonstrates the caloric contributions by and foraging efficiency of females. Our findings emphasize that child‐carrying techniques using basketry cradles allow for improved efficiency in foraging returns compared to more commonly used slings. Our results reinforce previous findings of female foraging efficiency despite the energetic demands of infant carrying and emphasize the energetic contributions of females to human evolution even during child‐rearing.

## 1 Introduction

The idea that there was a strict sexual division of labor in our evolutionary past has persisted within the field of anthropology for decades. The expression “man the hunter, woman the gatherer” is strongly embedded in both the mainstream and the academic understanding of the social hierarchies and associated responsibilities of our ancestors and many modern subsistence populations (Dahlberg 1981; Lee and DeVore 1968). By hunting big game, males are characterized as being the primary providers of calories, while females, splitting their time between child‐rearing and foraging, provide only minimal energetic contributions to the group. While highly debated now, these supposed differences in social and caloric contributions between the sexes were once depicted as the catalyst for human evolution and largely remain the source of stereotypical expectations of labor between males and females today (Crittenden et al. 2013; Kaplan et al. 2000; Lancaster and Lancaster 1983; Ocobock and Lacy 2023; Panter‐Brick 2002; Wrangham et al. 1999). Males are perceived as the primary providers because of the high energetic and nutritional value of meat acquired from hunting. However, given the unpredictability of hunting success, calories from hunting represent only a partial contribution to group intake (Hardy et al. 2022). Accumulating evidence sheds new light on the energetic contributions of foraging. Among living hunter‐gatherer populations females provide as much as 75% of caloric contributions by focusing on reliable food sources and rely little on resources from males to provide for themselves and their children (Marlowe 2001, 2007; Bliege Bird and Bird 2008). Among the Hadza, a modern hunter‐gatherer population from Tanzania, children, who were long believed not to contribute to the group's energetic needs, collect considerable amounts of food for themselves and the village (Crittenden et al. 2013). Alloparents and children, especially young girls, provide further support by caring for infants allowing mothers to increase their foraging rates during the postpartum period (Bove et al. 2002; Crittenden and Marlowe 2013; Jang et al. 2022; Meehan et al. 2013). The high nutritional value of foraged resources such as fruit, seeds, roots, nuts, shellfish, or even insects likely provide substantial energy to the forager and the overall group (Barlow and Heck 2002; Lesnik 2018). The high foraging return rates surpasses the caloric contributions of the rarer meat consumption from hunting.

While a substantial yet often overlooked body of work highlights the energetic contributions of mothers despite the biomechanical limitations of infant carrying (Kramer 1998; Prado‐Nóvoa et al. 2020; Wall‐Scheffler 2022), few have examined the energetic demands faced by mothers foraging with breastfeeding offspring and the behavioral and technological adaptations employed to attenuate the possible associated trade‐offs (Ross 2001; Wall‐Scheffler et al. 2007). Given the importance of maternal investment in altricial offspring and women's foraging contributions, these energetic trade‐offs and adaptations are critical to understanding our species' evolutionary past.

The present exploratory study aims to assess the energetic gain versus expenditure of female foraging and examines the efficiency of different child‐carrying techniques. Existing studies on total energy expenditure (TEE, kcal/day) and daily physical activity levels among different hunter‐gatherer populations demonstrate that daily energy expenditure is significantly greater for males compared to females in both the Shuar, a modern hunter‐gatherer population from Bolivia, and the Hadza (Christopher et al. 2019; Pontzer et al. 2012). While sex differences in energy expenditure among the Shuar are likely associated with aspects of their lifestyle, such as high physical activity levels and unique dietary patterns (Christopher et al. 2019), little work focuses on the energetics of specific tasks, limiting our understanding of their contributions to daily energy expenditure. Much of foraging by females, for instance, is done while either pregnant or rearing for their own or others' children, adding additional energetic demands (Hill and Kaplan 1999; Hrdy 2009; Panter‐Brick 1989).

Among many Indigenous communities in western North America, females contributed a substantial quantity of calories (approximately 60%–80%) to the household economy and, if reproductive, typically resumed foraging activities after a 2–6 week period of postpartum rest and/or seclusion (Bettinger 2015; Bibby 2004; Greenwald 2017; Kroeber 1925). Mothers carried their infants with them using basketry cradles or cradleboards unique to North America, rather than the more typical slings found worldwide (Greenwald 2017).

The present study simulates the foraging conditions of many precolonial Indigenous groups in California, where acorns (*Quercus*) were the primary staple food throughout the mid‐ to late‐Holocene and remain a culturally significant food to tribes today (Bettinger 2015; Kroeber 1925; Lightfoot and Parrish 2009). Indigenous Californians employed basketry cradle technology to safely transport infants; mothers carried infants and toddlers ranging from 1 month to 2 years of age to their acorn foraging or processing location and were able to safely set the baby down while working (Bibby 2004; Greenwald 2017; Shanks 2006, 2010, 2015). By leaning the cradled infant upright against a tree or rock, hanging from a tree, or staked upright in the ground, women were able to work unburdened with their infants safely within view and remain available for breastfeeding on demand (Greenwald 2017). In contrast, sling carrying, while still a very effective carrying strategy, adds weight to the torso, which can limit range of motion and limit certain movements such as bending at the hip and leaning forward (Hurtado et al. 1992; Greenwald 2017).

**FIGURE 1:**
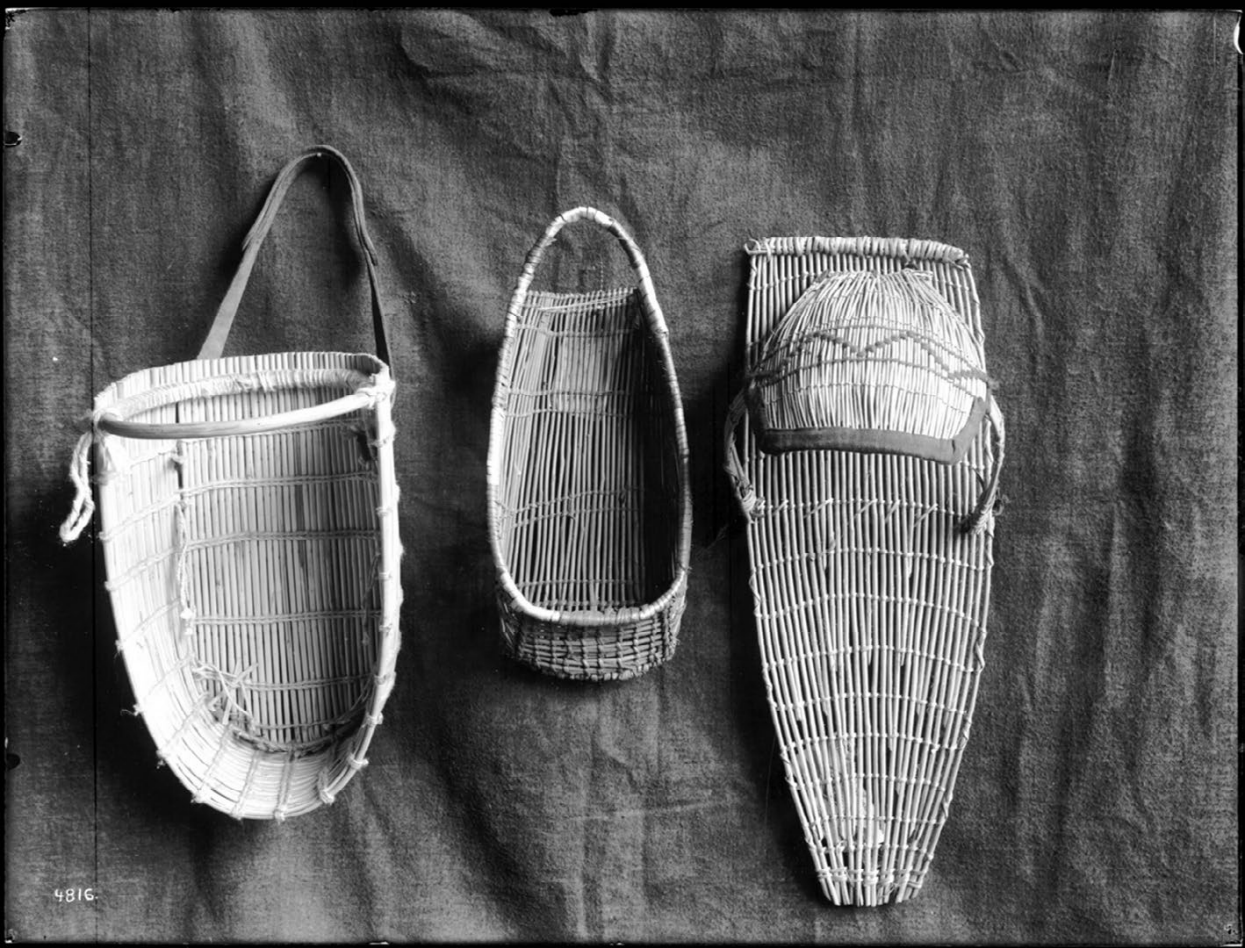
Three primary types of basketry cradles employed by Central Californians and Numic peoples of the Great Basin, including (L to R) the deep sitting cradle, the toe or slipper sitting cradle (used in this study), and the kite frame style of laying cradle. Photograph by Charles C. Pierce (1900), in the public domain.

The ubiquity of basketry cradles and cradleboards (see Figure 1) across groups in North America that relied heavily on women's contributions to the subsistence economy suggests the technology may be attenuating the energetic demands faced by foraging mothers (Greenwald 2017). We hypothesize that cradle technology increases caloric returns for foraging mothers by increasing their efficiency relative to foraging with a sling. We predict the mechanism of this increased efficiency is greater work capacity associated with the ability to safely “park” a cradled infant, as opposed to foraging while carrying the infant at all times.

This exploratory work aims to determine the differences in energetic output and gains between treatment groups foraging for acorns—females foraging without a load, foraging with an infant carried in a sling, and foraging with an infant carried in a traditional basketry cradle. By reproducing postpartum foraging situations in a small cohort of female participants, this work seeks to shed light on the energetic and social advantages of foraging, the energetic value of women's work, and the unique technology developed by Indigenous North Americans that may have alleviated maternal energetic demand and activity time allocation.

## 2 Materials and Methods

### 2.1 Study Sample and Design

Participants were recruited through flyers advertising the study on the University of Utah campus in Salt Lake City. Females between the ages of 18–40 years old in overall good health and able to walk for an hour carrying a 4.5 kg load were invited to participate. To limit the effects of confounding variables contributing to energy expenditure in this small exploratory study, pregnant and lactating individuals, as well as males were excluded from the study. The study was approved by the University of Utah and the University of Notre Dame IRB boards (proposal ID 22‐08‐7368). All participants provided written informed consent prior to enrolling in the study.

**FIGURE 2:**
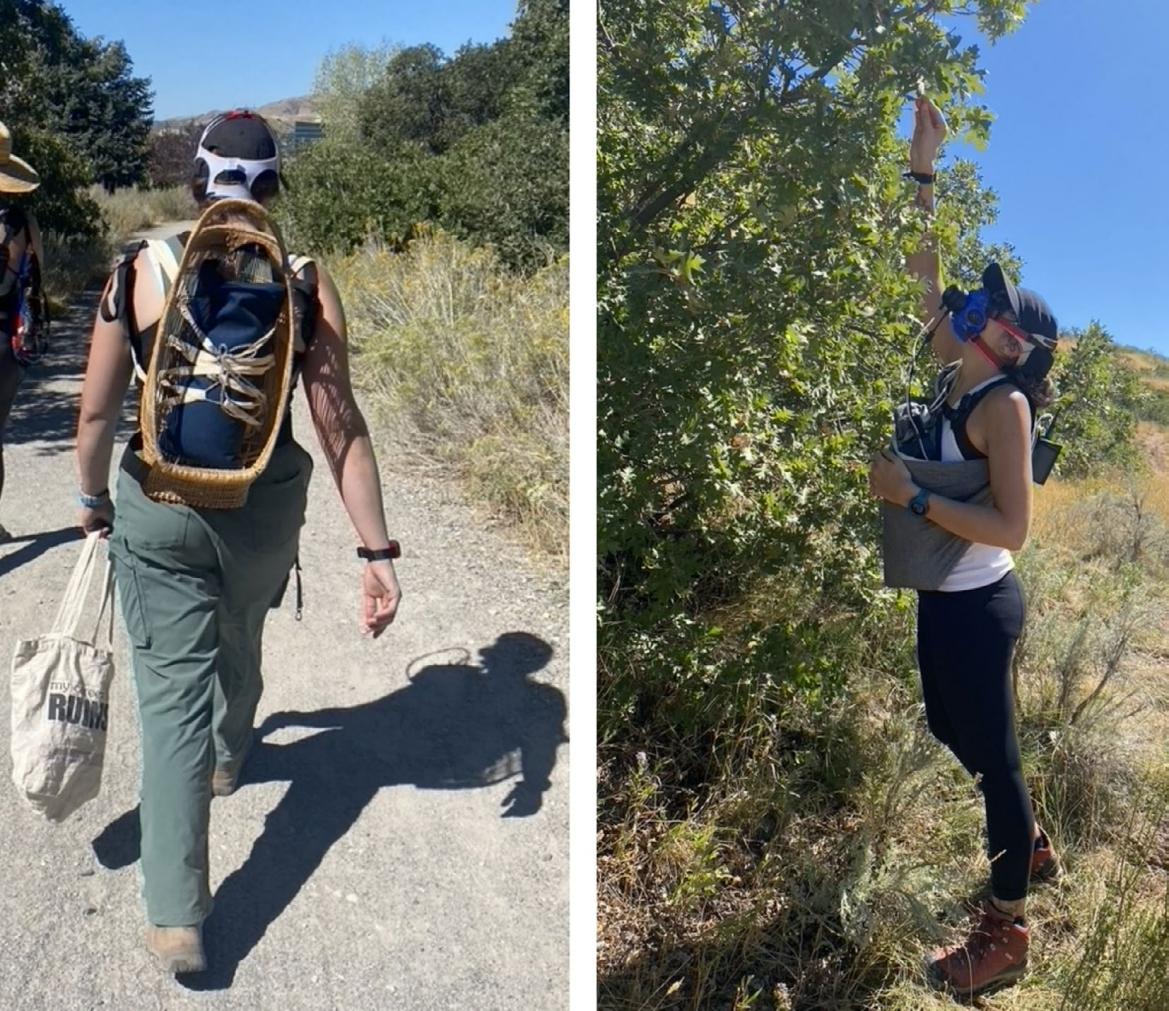
Cradle and sling as worn by participants using a 10lbs sandbag.

Participants (*n* = 6) arrived at the Department of Anthropology at the University of Utah at 8 a.m., being 12 h fasted. Anthropometric and resting metabolic rate measurements were collected. Several hours later that same day, with fasting requirements dropped, participants met the research team at the Natural History Museum of Utah, where they were provided with cloth bags and guidelines on acorn foraging on the Wasatch Front. Energetic and physical activity data were collected for three 1‐h bouts of acorn gathering at Gamble Oaks Grove. All bouts were conducted in groups of three participants. During the first bout, participants gathered acorns unloaded, meaning that they solely carried the portable indirect calorimetry unit (0.9 kg) and the cloth bag for acorn collection. Participants were then fitted for a second and third 1‐h foraging bout with, in a randomized order, a cross‐body sling or a basketry cradle, a traditional North American infant‐carrying technology, each containing a 4.5 kg sandbag mimicking the weight of a newborn infant (see Figure 2). All measurements started at an initial location < 0.5 miles from the foraging site to best encompass the energy expenditure for the entirety of the activity, including a short hike to the nearest oak trees. This also allowed participants to familiarize themselves with the equipment and breathing apparatus before foraging started. At the foraging site, the cradle was placed on the ground where it always remained in the participants' view, mimicking the “parking” of the cradle containing a secured newborn infant while allowing for the mother to keep an eye on her child during acorn gathering. For the sling condition, participants wore the sling holding the imitation infant when bending or leaning forward. New cloth bags for the collection of acorns were provided between each bout, and participants were free to move to different oak trees to maximize foraging results.

### 2.2 Anthropometric Measurements

Prior to the start of the foraging sessions, participants' height, weight, and body composition were measured following standard protocol (Lohman et al. 1988). Height was measured to the nearest millimeter using a tape measurer, while weight was measured to the nearest gram with an electronic scale (Taylor Precision Scale 7358T). Percent body fat (%BF), skeletal muscle mass (SMM), and fat‐free mass (FFM) were measured with a Quantum V Segmental bioelectrical impedance analyzer (RJL Systems, Clinton, MI) using preset standard NHANES‐III equations.

### 2.3 Energy Expenditure, Substrate Metabolism, and Activity

Participants arrived at the University of Utah Department of Anthropolgy after fasting for 12h. They rested on a cot in a supine position for 30 min. Resting metabolic rate (RMR, kcal/day) was then measured for an additional 30 min in the same position using a portable K5 indirect calorimetry unit (Cosmed, Chicago, IL). After individual calibration, the K5 unit measures O_2_ consumption and CO_2_ production by breath‐by‐breath analysis by using a mask with bi‐lateral unidirectional valves sealed around the participant's mouth and nose. A mask was used instead of a potentially more accurate hood for resting metabolic rate measurements as it allows for direct comparison of energy expenditure measurements collected during foraging bouts using the same K5 unit and mask.

With fasting requirements lifted, participants were fitted with the same mask around their mouths and noses during the acorn foraging bouts. For the first bout, the portable calorimetry unit was strapped, using the proprietary K5 chest harness (Cosmed, Chicago, IL), to participants' backs with the breathing mask fitted around their mouths and noses. For the short walk to and from the foraging site (< 0.5 miles), cradle‐carrying participants wore the calorimetry unit on their chests to fit the basket on their backs. The calorimetry unit was then switched to the back to match the position of the unit during sling and unloaded bouts prior to the start of foraging and after the cradle was safely placed on the ground (Figure 2). Energy expenditure was calculated for a total of 1 h during the initial “unloaded” acorn foraging bout, with no other loads being carried (EE_U_, kcal/h). After 15 min of rest and rehydration, energy expenditure was measured again in the same fashion for the 1‐h sling‐carrying, with the 4.5 kg load carried in a sling strapped to the chest, bout (EE_S_, kcal/h), and the 1‐h cradle‐carrying bout with the same load in a traditional cradle basket worn on the back (EE_C_, kcal/h). Participants carried their own cloth totes in which acorns were collected.

Participants wore an Actigraph wGT3X‐BT combination accelerometer‐heart rate monitor (Actigraph, Pensacola, FL) on their wrists with the associated heart rate chest strap during each foraging bout. Actigraphs were programmed to collect data at 30 s intervals for the three 1‐h foraging bouts. Activity intensity levels for each foraging bout recorded by the Actigraphs were used in data analysis. Participants wore the Actigraph monitors on their dominant hand, which they were most likely to use when plucking acorns from the trees. This allows for a fuller representation of their activity during foraging. Exercise intensity was determined using the ActiLife 6 software (version 6.3.4), which uses the default vector magnitude count based on the Freedson thresholds equation for adults (Freedson et al. 2011).

### 2.4 Acorn Foraging Returns

To calculate foraging returns, acorns were dried in screens separated by foraging bout. Cupules attached to the top of the acorn were removed, and the acorns were carefully shelled. Pre‐ and post‐sorted weights for both shells and nuts from all 18 foraging bouts were recorded. After shelling and weighing, a subsample of acorns was sent to the Central Analytical Laboratory at the University of Arkansas Poultry Science Center to perform bomb calorimetry, a method that involves measuring the heat generated by the combustion of the acorn sample, providing a direct measure of its caloric content. *Quercus gambelii* (Gamble Oak) acorns yield 3979 kcal/kg (Kievman et al. Forthcoming).

To calculate the average caloric value of the foraged acorns, we measure the net weight of a random sample of 120 acorns. To ensure a representative sample, single bouts worth of acorns are spread evenly across a screen, creating four quadrants. Thirty acorns are randomly selected from each quadrant to minimize bias and ensure that the sample accurately reflects the overall population of foraged acorns. We then extrapolate the nut weight using the nut ratio, which is calculated as the nut weight (g) divided by the sum of the nut weight (g) and shell weight (g). The nut ratio is then multiplied by the post‐sorted weight and caloric value. The caloric value (per kg) of each sample is determined by multiplying 3.979 (the caloric content of one gram of acorn) by the edible weight of all nut meat gathered in the foraging bouts.

$$\begin{array}{c} \mathrm{Nut}\;\text{weight}\;g/\left(\text{shell weight}\;\left(g\right)+\mathrm{nut}\;\text{weight}\;\left(g\right)\right)\\ =\mathrm{nut}\;\text{ratio}*\text{post}-\text{sorted weight}*\text{caloric value} \end{array}$$

$$\begin{array}{c} \text{The caloric value}\;\left(\mathrm{per}\;\mathrm{kg}\right)\;\text{of each sample}\\ =3.979*\text{edible weight for}\;\mathrm{all}\;\mathrm{nut}\;\text{meat gathered in bout} \end{array}$$

### 2.5 Statistical Analyses

Statistical analyses were performed in *RStudio* (version 1.2.533). Due to the small number of participants, statistical analyses were limited. Results are presented as min–max and means ± standard deviation. Variables were tested for normal distribution by plotting them in histograms.

Outcome variables such as EE, return rates (RR) from acorn collection, and foraging efficiency were compared between groups using repeated measures ANOVA. Foraging efficiency was measured as RR/EE.

## 3 Results

### 3.1 Descriptive Statistics

Descriptive statistics for all six participants are displayed in Table 1. All participants were in overall good health and were neither pregnant nor lactating.

**TABLE 1:** Individual descriptive statistics for age and anthropometric variables.

	Age (years)	Height (cm)	Weight (kg)	%BF	FFM (kg)	SMM (kg)
Subject 1	21	175.3	76.0	43.8	48.3	19.9
Subject 2	26	153.0	55.4	38.4	34.2	17.0
Subject 3	31	164.5	63.7	26.9	46.6	23.1
Subject 4	25	171.5	74.4	32.4	50.3	25.7
Subject 5	25	165.5	75.3	40.5	44.8	20.9
Subject 6	37	170.0	62.9	29.11	44.6	19.8
Averages	27.5 ± 5.7	166.6 ± 7.8	68.0 ± 8.5	35.2 ± 6.7	44.8 ± 5.6	21.1 ± 3.0

*Note:* Sample means and standard deviations for each variable are displayed separately.

Abbreviations: %BF, body fat percentage; FFM, fat‐free mass; SMM, skeletal muscle mass.

### 3.2 Energy Expenditures

**FIGURE 3:**
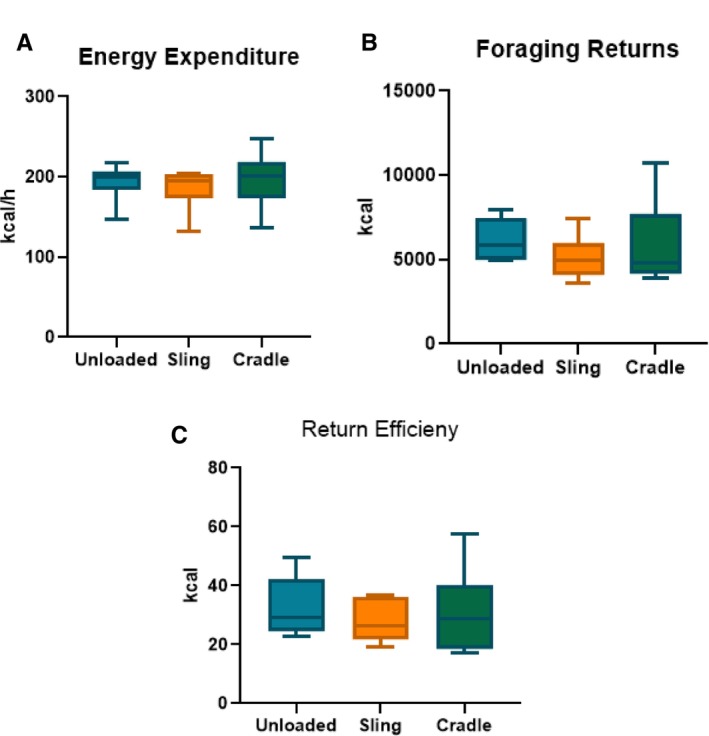
(A) Average energy expenditure for the unloaded (EE_U_), sling (EE_S_), and cradle (EE_C_) bouts. (B) Average caloric returns from acorn foraging for the unloaded (EE_U_), sling (EE_S_), and cradle (EE_C_) bouts. (C) Average return efficiency (energy expenditure/foraging returns) for the unloaded (EE_U_), sling (EE_S_), and cradle (EE_C_) bouts.

Table 2 displays RMR (kcal/day) as well as hourly energy expenditure (kcal/h) for each foraging bout by individual subject. Sample mean ± standard deviation for RMR, EE_U_, EE_S_, and EE_C_ are also displayed in Table 3. RMR measurements ranged between 1097 and 2028 kcal/day and foraging EE ranged from 132 to 247 kcal/h across all three bouts. Average EE for the sling‐carrying foraging bout was 4.12% greater than unloaded EE. EE_C_ was 1.55% greater than EE_U_ and 5.58% greater than EE_S_ (Figure 3A). Participants burned 213% more calories during the unloaded foraging bout, 200% more calories during the sling‐carrying bout, and 218% more calories during the cradle‐carrying bout compared to resting state. No significant differences in EE were found between bouts (repeated measures ANOVA: *p* = 0.41).

**TABLE 2:** Individual participant resting metabolic rate and hourly energy expenditures for the unloaded, sling‐, and cradle‐carrying foraging bouts.

	RMR (kcal/day)	RMR (kcal/h)	EE_U_ (kcal/h)	RR_U_ (kcal/h)	EE_S_ (kcal/h)	RR_S_ (kcal/h)	EE_C_ (kcal/h)	RR_C_ (kcal/h)
Subject 1	1527	64	200	7940	187	4245	209	4935
Subject 2^a^			147	7286	132	4767	137	4701
Subject 3	1097	46	203	5091	188	3586	198	6710
Subject 4	2028	85	218	4941	203	7430	205	3873
Subject 5	1666	69	196	4995	203	5523	247	4252
Subject 6	1138	47	199	6596	204	5193	186	10,734
Averages	1491 ± 387	62 ± 16	194 ± 24	6142 ± 1312	186 ± 28	5124 ± 1323	197 ± 36	5868 ± 2577

*Note:* Sample means and standard deviations for RMR and EE for each foraging bout are displayed separately.

Abbreviations: EE_C_, cradle energy expenditure; EE_S_, sling energy expenditure; EE_U_, unloaded energy expenditure; RMR, resting metabolic rate; RR_C_, cradle return rate; RR_S_, sling return rate; RR_U_, unloaded return rate.

^a^ RMR was not recorded for subject 2.

**TABLE 3:** Individual participant percentage of activity spent at light and moderate activity during each 1‐h foraging bout.

	Unloaded foraging bout		Sling‐carrying bout		Cradle‐carrying bout	
	Light intensity (%)	Moderate intensity (%)	Light intensity (%)	Moderate intensity (%)	Light intensity (%)	Moderate intensity (%)
Subject 1	44.2	55.8	27.8	72.2	31.1	68.9
Subject 2	74.7	25.3	68.4	31.6	18.9	81.1
Subject 3	27.5	72.5	4.6	95.4	13.9	86.2
Subject 4	18.3	81.7	20.8	79.2	7.7	92.3
Subject 5	26.4	73.6	5.0	95.0	11.5	88.5
Subject 6	14.3	85.7	9.2	90.8	13.3	86.7
Mean	34.2 ± 22.3	65.8 ± 22.3	22.6 ± 24.2	77.4 ± 24.2	16.1 ± 8.2	83.9 ± 8.2

*Note:* Sample means and standard deviations for light and moderate activity intensity percentages for each foraging bout are displayed separately.

### 3.3 Foraging Returns

The average caloric return for participants foraging without a load was 6142 kcal/h. When foraging with the “infant” carried in a cradle, the mean caloric return was 5868 kcal/h. The mean RR while foraging with a sling‐carried “infant” was 5124 kcal/h. Average return rates for each treatment group, as well as return rates for each participant across treatment groups, are reported in Table 2 and Figure 3B. These differences do not reach significance (*p* = 0.66), likely due to the small sample size.

Foraging efficiency, or the ratio of calories gained to calories expended, is reported in Figure 3C. The mean foraging efficiency for individuals gathering without a load is 33 kcal. Average foraging efficiency when foraging with cradle technology is 31 kcal, and 28 kcal when using a sling. As with variation observed in return rates, these differences do not reach significance (*p* = 0.75), likely due to the small sample size.

### 3.4 Physical Activity

Table 3 displays the percent of time spent in light and moderate intensity activity for each foraging bout and for each participant. Mean sample percentage spent at either light or moderate intensity ± standard deviation for each bout is also displayed. During all three 1 h‐bouts, most of it was spent at moderate intensity, with most time spent at moderate intensity during the cradle‐carrying bout. The sling‐carrying foraging bout, while on average over 70% of the time was spent at moderate intensity, had the greatest average percentage of light intensity activity (22.63%). Participants did not reach vigorous activity intensity levels during either foraging bout.

## 4 Discussion

Despite the small sample size, this exploratory study offers a promising look at maternal foraging energy expenditures and variation in foraging efficiency dependent on infant carrying technology. While not statistically significant, our findings suggest that foraging with a cradle as opposed to a sling yields slightly higher average caloric returns during acorn foraging. Transporting “infants” in cradles while foraging expended more calories, likely due to increased work capacity, but this higher‐intensity effort yielded greater returns. This dynamic lends support to our hypothesis that cradle technology increased foraging efficiency for mothers.

The utility of cradle carrying is not only reflected in the increased return rate of the method, but it is also emphasized by its rapid expansion across western North America after the prehistoric development of the technology among Basketmaker peoples in the Southwest (Greenwald 2017). Cradle carrying allows females to safely put down infants and regain full mobility to forage while keeping an eye on their offspring. The greater range of movement in turn allows for greater foraging intensity, resulting in increased energy expenditure but also increased foraging returns compared to the more restrictive sling‐carrying method, as it is rarely energetically advantageous to carry one's child versus setting them down and periodically checking on them (Kramer 1998). Females carrying their child across the chest in a sling expend less energy as their movements are limited by the sling and they are likely to decrease sudden or rapid movements to avoid disturbing the infant. Yet, sling‐carrying reduces the energetic and biomechanic burden of carrying an infant in one's arm, increasing the energetic value of sling carrying overall (Wall‐Scheffler et al. 2007).

The greater exertion observed during cradle‐carrying bouts is compensated for by the energetic returns from the acorns collected. The additional energy spent after securing the cradle and child on the ground is compensated for by higher energetic returns, increasing the efficiency of cradle compared to sling carrying. However, energy expenditure, energetic returns, and efficiency are higher in unloaded compared to cradle‐carrying foraging bouts. Despite having uninhibited mobility during foraging, individuals who use cradles carry them during the initial hike to the foraging location and periodically pause their foraging to check on their infant or move them as they progress to new locations during acorn collection, potentially contributing to decreased overall efficiency. Furthermore, cradle carrying required a switch in the placement of the portable calorimetry unit from the chest to the back in order to accommodate the cradle placed on the back during the short walk to and from the foraging site. Despite the relative brevity of the duration of the walk (approximately 5 min), this may have affected results as load placement can significantly impact energetic cost (Stuempfle et al. 2004).

In the present sample, cradle carrying results in the largest proportion of moderate intensity physical activity. This is of no surprise, as increased mobility during foraging, together with cradle carrying to and from, as well as between foraging locations, likely results in greater exercise intensity during the overall foraging bout compared to sling carrying or unloaded foraging. Sling carrying generated fewer bouts of moderate physical activity than cradle carrying, given the reduced mobility associated with this method, while simultaneously producing more frequent bouts of moderate physical activity compared to unloaded foragers, who may exert more energy during unrestricted foraging but who may do so at lower intensity levels, as they are not carrying extra weight in addition to their acorn gains. It is also important to note that while using the dominant wrist for accelerometry measurements to ensure the capture of activity during acorn picking, this may have overestimated physical activity.

Irrespective of the mode of infant carrying during foraging, this study empirically demonstrates the importance of maternal foraging contributions to hunter‐gatherer subsistence economies and undermines the notion that females of reproductive age rely primarily on male hunting efforts. The foraging returns are substantially larger than the energy expended over the course of the acorn collection periods. While the relative caloric contributions of male and female foragers are expected to vary between ecological zones, seasons, and the resources available within them, carrying and caring for infants do not dramatically inhibit women's capacity to gather a substantial quantity of food, even compared to males (Prado‐Nóvoa et al. 2020; Wall‐Scheffler 2022). Our results show that females can gather between 5124 and 6142 kcal on average per hour depending on their infant carrying method, underlining the significant caloric contributions by female foragers to the larger social group in a relatively short period of time. Furthermore, the choice to sling‐ or cradle‐carry infants during foraging is likely not limited by energetic outcomes and can be tied to environmental or social aspects facilitating the use of one carrying device over the other among different communities (Greenwald 2017; Wall‐Scheffler et al. 2007). The present paper, albeit exploratory, aligns with a growing body of work providing evidence for the sizable energetic and nutritional contributions of females across human evolution (Aiello and Key 2002; Goodman et al. 1985; Lacy and Ocobock 2023; Ocobock and Lacy 2023).

Despite these small steps in the right direction toward reconfiguring the misguided mainstream ideas about the role of females throughout evolution, more work is needed to address how the field of anthropology can support evidence‐based understanding and respect for contributions by females. Much of this work should continue to examine the contribution of females to calorie and nutritional provisioning and place specific emphasis on noting that bearing, rearing, or raising children does not limit these contributions (Crittenden et al. 2013; Crittenden and Marlowe 2013; Prado‐Nóvoa et al. 2020; Wall‐Scheffler 2022), though it may shape the resources that reproductive‐aged females pursue (Bliege Bird and Bird 2008).

### 4.1 Limitations

Future work, therefore, needs to take an intersectional approach when investigating the energetic contributions of humans across time and place. While small, this study provides an example of how comparing infant‐carrying methods as worn by modern‐day females can provide insight into the contributions of our ancestors. To expand on these findings, greater analytical power is needed by increasing the sample size. Limitations of this work include its cross‐sectional nature, which restricts our understanding of interindividual variability in energy expenditure and returns over time. Measuring loaded bouts after an initial unloaded bout may have affected results, as fatigue may have contributed to variations in energy expenditure and foraging efficiency. While the sample size limits controlling for baseline (unloaded) measures, participants reported that they were not fatigued after the first foraging bout given the milder temperatures early in the morning and the low exercise intensity of unloaded foraging. It is also important to note that while using the dominant wrist for accelerometry measurements to ensure the capture of activity during acorn picking, this may have overestimated physical activity. Furthermore, this work is limited in testing a specific evolutionary hypothesis on the contributions of female energetics to human evolution. What it does, however, is introduce new notions of the social and behavioral interactions during foraging that likely shaped evolutionary trajectories.

While this study did not include Indigenous study participants during the experimental stage, extensive ethnographic interviews with 20 elders, tribal leaders, and knowledge keepers from 11 tribal affiliations guided our methods of cradle use and foraging. We believe that it is of utmost importance to consider Indigenous perspectives, skill sets, and research questions when conducting work based on Indigenous traditions and lifeways. Indigenous insights also contribute to accurate depictions and conclusions by rooting empirical data findings in traditional knowledge and understanding of the mitigating effects at play during similar recreations. It is only by taking a truly intersectional approach to reframing our understanding of gender roles throughout human evolution, which includes female and Indigenous voices, that we will be able to garner the most accurate picture of our common past.

## Author Contributions

**Alexandra Niclou:** conceptualization (equal), data curation (equal), formal analysis (equal), investigation (equal), methodology (equal), project administration (equal), visualization (lead), writing – original draft (lead), writing – review and editing (equal). **Alexandra Greenwald:** conceptualization (equal), data curation (equal), formal analysis (equal), investigation (equal), methodology (equal), project administration (equal), resources (lead), supervision (equal), visualization (equal), writing – original draft (equal), writing – review and editing (equal). **Cara Ocobock:** conceptualization (equal), data curation (equal), investigation (equal), methodology (equal), project administration (equal), resources (equal), supervision (lead), writing – review and editing (equal).

## Conflicts of Interest

The authors declare no conflicts of interest.

## Data Availability

The data is available upon reasonable request to the corresponding author.
